# Pattern of antioxidant enzyme activities and hydrogen peroxide content during developmental stages of rhizogenesis from hypocotyl explants of *Mesembryanthemum crystallinum* L.

**DOI:** 10.1007/s00299-013-1520-4

**Published:** 2013-10-18

**Authors:** Robert Konieczny, Agnieszka K. Banaś, Ewa Surówka, Żaneta Michalec, Zbigniew Miszalski, Marta Libik-Konieczny

**Affiliations:** 1 Department of Plant Cytology and Embryology, Institute of Botany, Jagiellonian University, Gronostajowa 9, 30-387 Kraków, Poland; 2 Department of Plant Biotechnology, Faculty of Biochemistry, Biophysics and Biotechnology, Jagiellonian University, Gronostajowa 7, 30-387 Kraków, Poland; 3 The Franciszek Górski Institute of Plant Physiology, Polish Academy of Sciences, Niezapominajek 21, 30-239 Kraków, Poland

**Keywords:** Catalase, *Mesembryanthemum crystallinum*, Hydrogen peroxide, Peroxidase, Superoxide dismutase, Metabolism

## Abstract

*****Key message***:**

**H**_**2**_ **O**_**2**_ **is necessary to elicit rhizogenic action of auxin. Activities of specific catalase and manganese superoxide dismutase forms mark roots development.**

**Abstract:**

Hypocotyl explants of *Mesembryanthemum crystallinum* regenerated roots on medium containing 2,4-dichlorophenoxyacetic acid. Explants became competent to respond to the rhizogenic action of auxin on day 3 of culture, when hydrogen peroxide content in cultured tissue was the highest. l-Ascorbic acid added to the medium at 5 μM lowered the H_2_O_2_ level, inhibited rhizogenesis and induced non-regenerative callus, suggesting that certain level of H_2_O_2_ is required to promote root initiation. Coincident with the onset of rhizogenic determination, meristemoids formed at the periphery of the hypocotyl stele and the activity of the manganese form of superoxide dismutase, MnSOD-2 was induced. Once induced, MnSOD-2 activity was maintained through the post-determination phase of rooting, involving root growth. MnSOD-2 activity was not found in non-rhizogenic explants maintained in the presence of AA. Analyses of the maximum photochemical efficiency of photosystem II and the oxygen uptake rate revealed that the explants were metabolically arrested during the predetermination stage of rhizogenesis. Respiratory and photosynthetic rates were high during root elongation and maturation. Changes in catalase and peroxidase activities correlated with fluctuations of endogenous H_2_O_2_ content throughout rhizogenic culture. Expression of a specific CAT-2 form accompanied the post-determination stage of rooting and a high rate of carbohydrate metabolism during root growth. On the other hand, the occurrence of MnSOD-2 activity did not depend on the metabolic status of explants. The expression of MnSOD-2 activity throughout root development seems to relate it specifically to root metabolism and indicates it as a molecular marker of rhizogenesis in *M. crystallinum.*

## Introduction

Reactive oxygen species (ROS) such as superoxide radical (O_2_^·−^) and hydrogen peroxide (H_2_O_2_) serve as important signaling molecules in plant growth and differentiation (review by Ślesak et al. 2007). In rhizogenesis, ROS have been found to be involved in quiescent center formation (Joo et al. 2001), root elongation (Liszkay et al. 2004), root hair development (Foreman et al. 2003) and xylem differentiation (Tisi et al. 2011). Recently, Li et al. identified H_2_O_2_ as a downstream component in the auxin-mediated signaling pathway leading to root initiation in mung bean seedlings (Li et al. 2009a, b).

In all living cells, H_2_O_2_ is continually generated as a by-product of aerobic metabolism. Respiratory chain and photosynthetic electron transport are well-documented sources of this molecule in plants (review by Ślesak et al. 2007). The amount of H_2_O_2_ produced by mitochondria and chloroplasts depends on their metabolic activity, in that high respiratory and photosynthetic rates facilitate overreduction of electron carriers and direct flow of electrons to molecular oxygen, eventually giving rise to a rapid increase in cellular H_2_O_2_. ROS/H_2_O_2_ production also increases in response to abiotic stress associated with, for example, tissue culture (review by Bat’ková et al. 2008). Because of its high toxicity and due to its signaling function, the endogenous H_2_O_2_ level is tightly controlled by the coordinated action of H_2_O_2_-generating enzymes such as superoxide dismutases (SODs; EC 1.15.1.1) and H_2_O_2_-scavenging enzymes such as catalase (CAT; EC 1.11.1.6.) and peroxidases (POXs; EC 1.11.1.7).

Organogenesis in vitro is a stress-related and energy-consuming process which is accompanied by intensification of catabolic and anabolic reactions (Millar et al. 1998; Gaspar et al. 2002; Balen et al. 2009). Changes in endogenous H_2_O_2_ content as well as the differential activities of SODs, CAT and POXs accompany rhizogenesis in vitro in several species including oak Racchi et al. (2001), mung bean (Li et al. 2009a, b) or tomato (Tyburski et al. 2009). How the specific activity pattern of the antioxidant machinery and fluctuations of the H_2_O_2_ level are related to the respiratory/photosynthetic rates of cultured explants still needs clarification.

If the physiological changes during rhizogenesis are to be understood developmentally, they have to be tracked through the different stages of root ontogeny. Christianson and Warnick divided morphogenesis in vitro into three developmental stages: competence, determination and morphological differentiation (Christianson and Warnick 1983, 1985). Competence is the ability of cells to respond to an inductive stimulus; determination is an irreversible change in the physiology of an explant cell or group of cells which dictates a unique developmental fate. When determination has occurred, the determined cells continue to develop through the post-determination phase, eventually differentiating adventitious organs. The time needed for induction of morphogenic determination can be found experimentally by noting when, after exposure to an induction stimulus, an explant is able to produce organs/embryos in the absence of that stimulus. The temporal framework of organogenic/embryogenic competence can be defined by transferring explants from non-inductive to inductive media (Christianson and Warnick 1985). Explant-transfer experiments have been successfully employed to determine the series of stages in morphogenic processes of several species. Specific features of growth regulator metabolism, protein turnover, DNA biosynthesis and gene activity patterns during organogenic determination and/or acquisition of competence have been described in petunia, tobacco and *Arabidopsis* (Auer et al. 1992; Kim and Ernst 1994; Che et al. 2007). Not studied previously is the involvement of oxidative events in acquisition of rhizogenic competence and determination.

The common ice plant (*Mesembryanthemum crystallinum* L.) is a useful model for studying plant responses to different environmental stresses (Cushman and Bohnert 2000; Konieczny et al. 2011). The role of oxidative stress in morphogenesis in vitro has been examined in this species (Libik et al. 2005; Libik-Konieczny et al. 2012). Calli differing in regeneration potential (i.e., rhizogenic or embryogenic) also differed in H_2_O_2_ content as well as in SODs and CAT activities, suggesting the possible involvement of ROS in induction of different morphogenic pathways (Libik et al. 2005). In addition, a specific MnSOD form, referred to as MnSOD-2, was expressed in rhizogenic calli but never in embryogenic and or non-regenerative ones (Libik et al. 2005; Libik-Konieczny et al. 2012). MnSOD-2 activity was also detected in roots of *M. crystallinum* growing in vitro and was suggested to be induced by respiratory-stimulating conditions of tissue culture (Ślesak and Miszalski 2002). Moreover, based on biochemical studies Ślesak and Miszalski (2002) put into the question the SOD-like nature of MnSOD-2 and suggested that this protein may in fact represent germin-like protein (GLP) of SOD activity. In that report, however, the rate of carbohydrate metabolism in the MnSOD-2-expressing material, as well as putative germin-like nature of MnSOD-2, was not confirmed.

In this study, we characterized the anatomical changes and oxidative events (pattern of SODs, CAT and POXs activity, and changes in endogenous H_2_O_2_ content) during successive stages of root regeneration from *M. crystallinum* hypocotyl explants, and related them to rates of basal metabolism (respiratory and photosynthetic activity) at given stages of rooting. We analyzed SOD and CAT isoforms to find isoenzymes that were active in a phase-dependent manner. We demonstrated the importance of H_2_O_2_ in root initiation, and verified the putative germin-like nature of MnSOD-2 by tracking the expression of a gene encoding a root-specific germin-like protein in *M. crystallinum* (*McGLP* (Michalowski and Bohnert 1992). All biochemical and microscopic analyses presented here employed a newly established system for direct root regeneration which avoids the physiological instability associated with growth of callus.

## Materials and methods

### Plant material

Seeds of *Mesembryanthemum crystallinum* (L.) were obtained from plants grown in a phytotron chamber at 25/20 °C L/D under a 16 h photoperiod (250–300 μmol^−2^ s^−1^, Merazet KB + WF 720 tubes) at 65 % RH. Seeds were sterilized for 60 s in 70 % (v/v) ethanol and for 15 min in commercial bleach (Domestos, Unilever, Poland) diluted with water (1:2 v/v). Then, the seeds were rinsed three times with sterile distilled water and germinated on 9 cm Petri dishes filled with 15 ml basal medium (BM) composed of Murashige and Skoog salts and vitamins (Murashige and Skoog 1962) (Sigma, Germany), 30 g l^−1^ sucrose and 7 g l^−1^ agar (Difco Bacto, USA), pH 5.7. Dishes with seeds were kept in a growth chamber at 25/20 °C L/D under a 16 h photoperiod (100–120 μmol m^−2^ s^−1,^ Merazet KB + WF 720 cool-white tubes). After 10 days of germination, hypocotyl explants 5–7 mm long were excised from the seedlings and placed on culture media.

### In vitro culture conditions

To induce rhizogenesis, the hypocotyls were placed horizontally on root-inducing medium (RIM) consisting of BM supplemented with 1 mg l^−1^ 2,4-dichlorophenoxyacetic acid (2,4-D) (Sigma, Germany). To assess the involvement of H_2_O_2_ in root induction, hypocotyl explants were maintained on RIM supplemented with the antioxidant l-ascorbic acid (AA) (Sigma, Germany) at concentrations of 2.5 or 5 μM. The aqueous solution of AA was filter-sterilized and aseptically introduced to previously autoclaved and cooled medium. Explants maintained on BM were the control. Hypocotyls were cultured on BM, RIM or RIM + AA for 14 days and then the frequency of rhizogenesis (hypocotyls producing roots/total number of explants in each treatment × 100) and mean number of roots induced from hypocotyls (total number of roots/number of hypocotyls showing rhizogenesis) were calculated.

To determine the time of induction of rhizogenic competence and determination, reciprocal transfers were made between BM and RIM and between RIM and BM, respectively. This was done daily up to day 8 of culture. Root appearance was noted and qualitative data on regeneration (as above) were collected daily.

In all in vitro experiments, the explants were maintained in a phytotron chamber under the same conditions as described for seed germination. Each treatment involved 50 hypocotyls, 10 per Petri dish (9 cm diam., containing 20 ml medium).

### Histological studies

Material for light microscopy observations was collected daily from days 1 to 14 of continuous culture on RIM and on RIM with 5 μM AA. Fixation and embedding followed procedures described earlier by Konieczny et al. (2003). Sections (5 μm thick) from explants maintained on rhizogenic (RIM) and non-rhizogenic (RIM with 5 μM AA) medium were stained with 0.1 % (w/v) aqueous solution of toluidine blue (Sigma, Germany). Hypocotyls fixed without being grown on culture media were the control.

### Biochemical studies

Unless stated in the procedure for SOD activity studies, biochemical analyses were performed on material collected on days 0, 1, 3, 5, 7, 9 and 14 of continuous culture on RIM. Additionally, we analyzed non-rhizogenic explants maintained on RIM supplemented with 5 μM AA to examine the relationship between endogenous H_2_O_2_ content and the explants’ ability to produce roots, and also to verify the root-specific profile of SOD forms in material cultured on RIM.

### Protein isolation

To isolate fractions of soluble proteins, plant material (1 g fresh weight) was homogenized at 4 °C with a mortar in 2.5 ml homogenization buffer (17.9 g l^−1^ TRICINE, 0.74 g l^−1^ MgSO_4_, 0.155 g l^−1^ DTT, 1.14 g l^−1^ EDTA, adjusted with 1 mol l^−1^ TRIS to pH 8.0). Non-soluble material was removed by centrifugation for 3 min at 3,000*g*.

### Determination of protein content

The protein concentration was determined according to Bradford (1976) using the Bio-Rad Protein Assay Kit with BSA as standard. Protein fractions were stored at −80 °C until further use.

### Visualization of SOD activity on gel after native PAGE

Fractions of soluble proteins isolated from material collected on days 0, 1, 3, 5, 7 and 14 of continuous culture on RIM were separated by native PAGE at 4 °C and 180 V in the Laemmli buffer system (Laemmli 1970) without sodium dodecyl sulfate (SDS). SOD bands were visualized on 12 % polyacrylamide gels using the activity-staining procedure described by Beauchamp and Fridovich (1971): the gels were incubated in staining solution [potassium phosphate buffer, pH 7.8, containing 0.0068 g l^−1^ KH_2_PO_4_, 0.0175 g l^−1^ Na_2_HPO_4_, 0.372 g l^−1^ EDTA, 31 % (w/v) TEMED, 7.5 mg l^−1^ riboflavin and 0.2 g l^−1^ NBT] for 30 min in the dark at room temperature and then exposed to white light until SOD activity bands became visible.

### Visualization of CAT activity on gel after native PAGE

Fractions of soluble proteins were separated by native PAGE. CAT bands were visualized on 10 % polyacrylamide gels using the activity-staining procedure described by Woodbury et al. (1971): gels were incubated for 15 min in 0.03 % (v/v) H_2_O_2_ and then stained in 25 ml solution containing 20 g l^−1^ FeCl_3_ and 20 g l^−1^ K_3_Fe(CN)_6_.

### Spectrophotometric analysis

Catalase (CAT) activity was determined after protein isolation from 0.1 g tissue homogenized in 2 ml 300 mmol l^−1^ Tricine buffer, pH 8.0, containing 3 mmol l^−1^ MgSO_4_, 3 mmol l^−1^ EGTA and 1 mmol l^−1^ DTT. The extract was then centrifuged at 3,000*g* at 4 °C and the supernatant was collected. CAT activity was measured according to the method described by Aebi (1984). The disappearance of H_2_O_2_ [initial concentration: 0.04 % (v/v) H_2_O_2_] in phosphate buffer (50 mmol l^−1^ KH_2_PO_4_, 50 mmol l^−1^ Na_2_HPO_4_, pH 7.0) was monitored at 240 nm. Enzyme activity was defined as 1 mmol H_2_O_2_ decomposed per minute. Calculations used an absorbance coefficient of 43 l M^−1^ cm^−1^.

Guaiacol peroxidase (POX) activity was determined according to Pütter (1974) after homogenization of 0.1 g frozen tissue in 1 ml 300 mmol l^−1^ potassium phosphate extraction buffer, pH 7.0, containing 1 mmol l^−1^ EDTA. The extract was centrifuged at 10,000*g* at 4 °C for 3 min. The reaction was run for 5 min at 25 °C in a 1 ml cuvette with 50 μl purified extract in 300 mM potassium phosphate buffer, pH 6.1, in the presence of 8.42 mmol l^−1^ guaiacol and 2.10 mmol l^−1^ H_2_O_2_. Conversion of guaiacol to tetraguaiacol was monitored at 470 nm, and POD activity was calculated using the absorbance coefficient 26,600 l M^−1^ cm^−1^.

### Quantification of H_2_O_2_ concentration

Amplex Red H_2_O_2_/peroxidase assay kit was used for hydrogen peroxide level estimation. Hypocotyls (0.1 g) were grounded with a mortar in 0.5 ml of reaction buffer (provided in the kit). The extract was centrifuged for 5 min at 14,000×*g* and supernatant was collected for further analysis: 50 μl of the supernatant was incubated for 30 min at room temperature under dark conditions, with 50 μl of working solution containing 100 mM Amplex Red reagent and 0.2 units ml^−1^ horseradish peroxidase. Hydrogen peroxide concentration was then measured spectrofluorymetrically with a 96-well microplate reader Synergy 2 (Bioteck) using excitation at 530 nm and fluorescence detection at 590 nm. The standard curve in the range 0.05–1 μM was made using a different concentration of H_2_O_2_ provided in the kit.

### Densitometric analysis

For densitometric analysis of SOD activity, three gels from three independent experiments were analyzed. The gels were scanned and next analyzed using Bioprint ver. 99 (Vilaber-Lourmat, France). Total SOD activity is expressed in arbitrary units corresponding to the area under the densitometric curve.

### Measurement of photosynthetic activity—fluorometric method

The maximum photochemical efficiency of photosystem II (*F*_v_/*F*_m_) of chlorophyll *a* was measured with the FluorCAM imaging system (PSI, Brno, Czech Republic). Before the measurements, the explants were light-adapted under red–orange LED actinic light for 10 min and the maximum light-adapted fluorescence (*F*_m_) was measured (saturating flash). Analyses of chlorophyll *a* fluorescence parameters were done for each hypocotyl separately. Maximum efficiency of PSII (*F*_v_/*F*_m_) was calculated as described by Baker et al. (2001) and Schreiber et al. (1994).

### Measurement of respiration activity

Respiration activity was measured as the rate of oxygen consumption in liquid phase using a Clark oxygen electrode (Oxytherm, Hansatech Instruments, England). The electrode was calibrated with dithionite according to the manufacturer’s instructions. An initial oxygen concentration in the range 180–200 nmol O_2_/ml was taken as the starting point. Ten hypocotyls from each day of culture were suspended in 900 μl sterile deionized water and placed in the electrode chamber and covered with a dark lid. Samples were continuously mixed with a magnetic stirrer for homogenization of gas in the chamber. Oxygen consumption was measured for 20 min at 25 °C. The results were calculated with Oxygraph Plus software (Hansatech Instruments, England) and are expressed as nmol O_2_ min^−1^ mg fresh weight^−1^.

### Determination of explant growth rate

The rate of explant growth on RIM is expressed as the change in fresh weight (FW) on days 1, 3, 5, 7, 9 and 14 of culture. Fifty explants were weighed each day and the mean weight of a single explant was calculated. Explants before placing onto RIM were used as a control.

### RNA isolation, RT-PCR and semi-quantitative PCR

Total RNA was isolated from roots of 3-month-old plantlets and explants maintained on RIM for 1, 3, 5, 7, 9, and 14 days using TRI Reagent^®^ (BioChemika, Switzerland) according to the manufacturer’s protocol. DNA contamination was removed with RNase-free DNase I (Fermentas, Lithuania). First-strand cDNA synthesis was performed using a RevertAid™ First Strand cDNA Synthesis Kit (Fermentas, Lithuania) and primed with random hexamer primers. QuantumRNA™ 18S RNA (Ambion Europe Ltd UK) with a 3:7 primer:competimer ratio functioned as internal standard in simultaneous PCR amplification with primers specific for the gene coding M. crystallinum germin-like protein (forward: 5′-GCCACGACTCTCTATCAGG-3′; reverse: 5′-TCGAAGGCCTTAGCAAGAAC-3′).

The PCR amplification with RUN polymerase (A&A Biotechnology, Poland) was carried out with a Bio-Rad MJ Mini Gradient Thermal Cycler using the following program: 94 °C for 30 min, the 28 cycles at 94 °C (30 s), 50 °C (15 s), and 72 °C (40 s), followed by a final 3 min 72 °C incubation.

### Statistical analysis

For each experiment, the means of five (in vitro culture, determination of FW) or three (biochemical analyses) replicates were calculated. The experiments were repeated three times. Statistical differences between means (*p* ≤ 0.05) were determined by two-way ANOVA followed by Tukey’s multiple range test using Statistica for Windows ver. 8.0 (StatSoft, Inc., Tulsa, Oklahoma, USA).

## Results

### 2,4-D induces direct rhizogenesis from hypocotyl explants

**Fig. 1:**
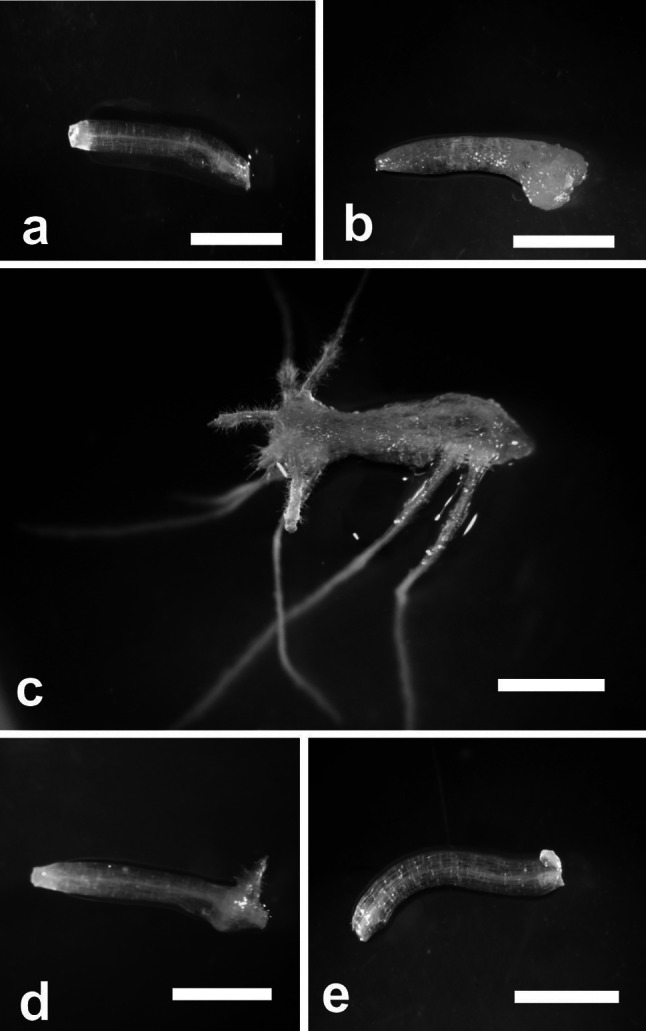
Hypocotyl explants cultured on different media. Hypocotyl explant before culture (**a**), at day 14 of culture on BM (**b**), at day 14 of culture on RIM (**c**), at day 14 of culture on RIM + 2.5 μm AA (**d**), at day 14 of culture on RIM + 5 μM AA (**e**). *Scale bar* 0.2 mm

The presence of auxin in the medium was necessary for root initiation. Hypocotyl explants (Fig. 1a) did not produce roots after 14 days of continuous culture on BM, instead of that they became swollen at the cut end and formed small amounts of non-regenerative callus (Fig. 1b). The explants maintained on RIM started to produce roots 9 days after explantation. Rhizogenesis was direct throughout the culture, with means of 94 % frequency and 4.3 roots/explant at day 14 (Fig. 1c).

### Rhizogenic determination occurs at day 5 of culture

**Fig. 2:**
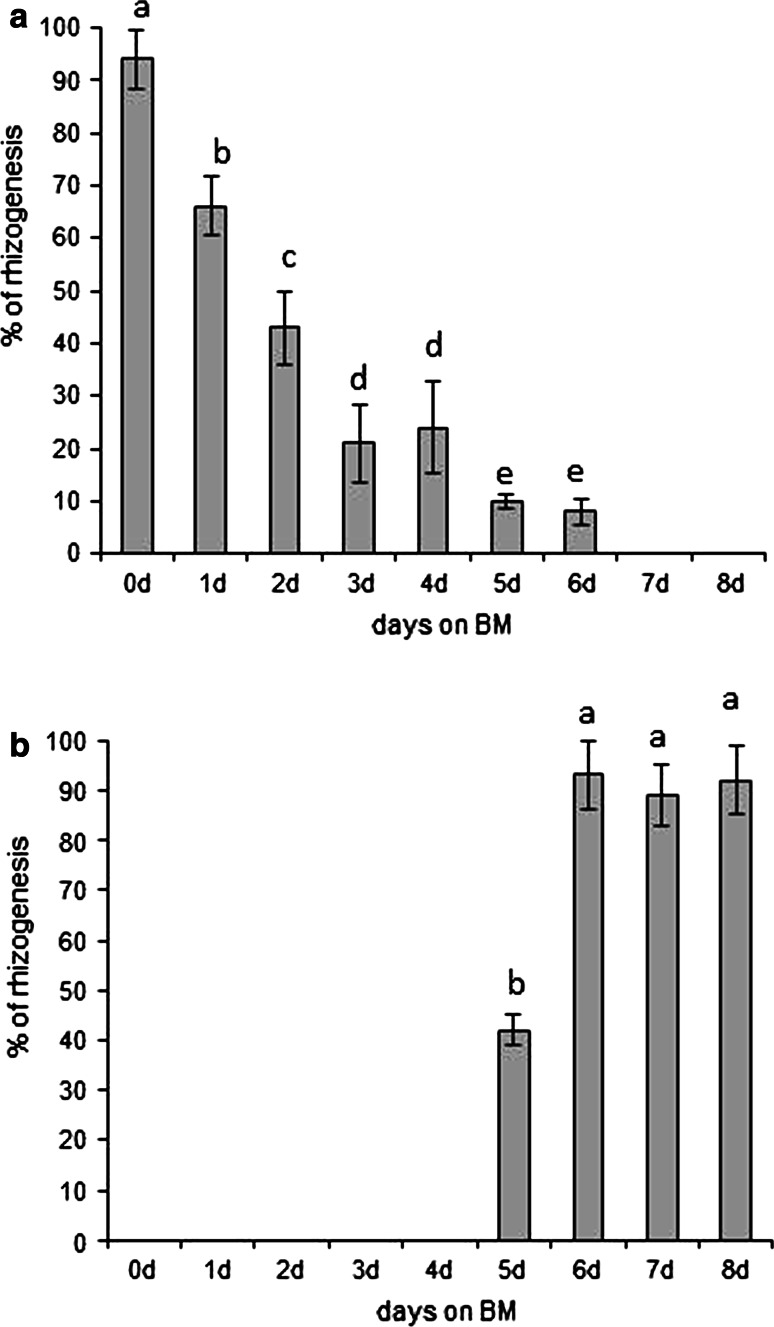
Frequency of rhizogenesis (%) during explant’s culture on BM (**a**), or on RIM (**b**) for various periods of time before transfer to RIM or BM, respectively. Values sharing the same letter are not significantly different (*p* ≤ 0.05) according to Tukey’s multiple test

The duration of culture on BM before transfer to RIM had an obvious effect on the percentage of responding explants, the number of roots produced, and the time needed for the first root to appear. When hypocotyls were precultured on BM for 1–6 days before transfer to RIM, the period before the first root appeared on RIM was shortened by 1–3 days depending on the time on BM (Table 1). Longer preculture on BM caused the hypocotyls either to produce non-rhizogenic callus or to decay when subcultured on RIM. Increasing the time of preculture on BM greatly reduced rhizogenesis frequency (Fig. 2a). After 6 days on BM, only a few explants regenerated single roots on RIM, and no roots were obtained on RIM when preculture on BM was prolonged to 7 or 8 days.

**Table 1:** Number of days for first root appearance from hypocotyls of *M. crystallinum* on RIM after preculture on BM for 0–8 days

	Days of preculture on BM								
	0	1	2	3	4	5	6	7	8
Day of first root appearance	9	8	7	6	6	6	7	–	–

Transfers from RIM to BM revealed that 5-day exposure to RIM was required to elicit rhizogenesis on BM (Fig. 2b). The regeneration percentage increased up to day 6 of culture. Further culture on RIM prior to transfer had no effect on rhizogenesis.

### Meristemoids formation is observed at day 5 of culture

**Fig. 3:**
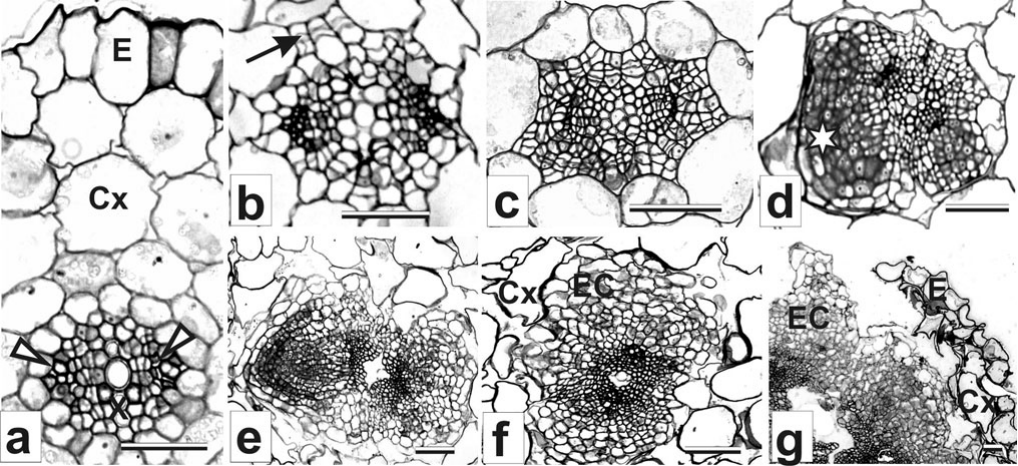
Transverse sections through initial explant and explants maintained on RIM and on RIM with 5 μM AA. **a** Hypocotyl explant before culture. **b** Induction of meristematic activity at day 1 of culture on RIM. **c** Intense cell proliferation within vascular cylinder at day 4 of culture on RIM. **d** Production of meristemoid at day 5 of culture on RIM. **e** Root primordium at day 7 of culture on RIM. **f** Production of endogenous callus at day 5 of culture on RIM with 5 μM AA. **g** Rupture of epidermis and outgrowth of callus over explant surface at day 10 of culture on RIM with 5 μM AA. *Cx* cortex, *E* epidermis, *EC* endogenous callus, *X* xylem. *Arrowheads* on **a** indicate phloem pools; *arrow* on **b** indicates periclinal divisions of innermost cells of cortex; *asterisk* on **d** indicates meristemoid. *Scale bar* 50 μm

Sections through freshly cut hypocotyls revealed a single-layered epidermis, 4–5 layers of cortex, and a centrally located stele with two strands of cambium extending between xylem and phloem pools (Fig. 3a). Some meristematic activity was seen in the central part of the hypocotyl as soon as day 1 on RIM. These cell divisions were predominantly periclinal and localized to phloem pools. Single dividing cells in the inner layer of cortex were observed occasionally (Fig. 3b). With continued culture the cortical cells divided only sporadically, while intense cell proliferation was observed within the entire stele (Fig. 3c). Five days after explantation masses of small, densely stained cells formed regularly into meristemoids at the stele periphery (Fig. 3d). The meristemoids became polarized and differentiated into typical cone-shaped root primordia by day 7 on RIM (Fig. 3e). The cells of the epidermis and external layers of cortex were not involved in root formation; with culture they enlarged enormously, became detached from each other and degenerated.

### Increase in H_2_O_2_ accumulation is noted at early stages of rhizogenesis

**Fig. 4:**
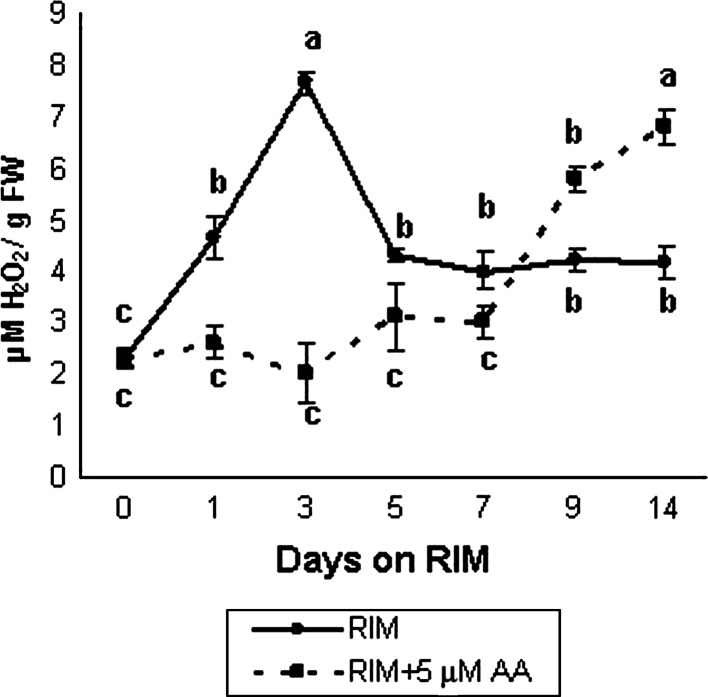
Endogenous concentration of hydrogen peroxide in explants maintained on RIM and RIM with 5 μM AA for 14 days. Values on graph represent mean ± standard error (SE).Treatments bearing the *same letter* do not differ significantly (*p* ≤ 0.05) by Tukey’s multiple range test

In explants maintained on RIM, H_2_O_2_ content increased gradually in early stages of culture and peaked on day 3 when it was about 3 times higher than in hypocotyls before explantation (Fig. 4). Then, the level of hydrogen peroxide decreased in explants cultured on RIM within 7–14 days; however, it was not lower than in initial explants.

### Induction of specific formes of SOD and CAT and increase in POX activity during rhizogenesis is observed

**Fig. 5:**
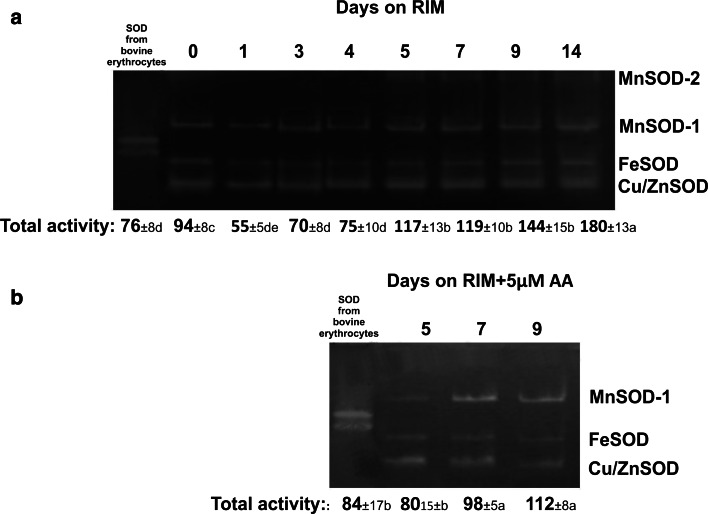
Representative gels showing staining for SOD activity following native PAGE of protein extracts from explants maintained for 14 days on RIM (**a**) and on RIM with 5 μM AA (**b**). Each well was loaded with 15 μg proteins. SOD from bovine erythrocytes (6,615 units/mg protein, Sigma–Aldrich) was loaded as a control. Numbers below gels indicate the activity of SOD forms (expressed in arbitrary units—area under densitometric curve). Treatments bearing the *same letter* do not differ significantly (*p* ≤ 0.05) by Tukey’s multiple range test

On the basis of sensitivity to KCN and H_2_O_2_, three well-visible bands of SOD activity were identified in material from explants before placing on RIM: Cu/ZnSOD (fast-migrating band), FeSOD (band of intermediate mobility) and MnSOD-1 (slower-migrating band) (Fig. 5a). The Cu/ZnSOD, FeSOD and MnSOD-1 bands were observed throughout culture on RIM. At day 5, an additional band of MnSOD-2 activity appeared. Once induced, the MnSOD-2 band was also detected at 7, 9 and 14 days of culture. Densitometric analysis (Fig. 5a) showed that total SOD activity decreased during the first 4 days of culture. At 5 days of culture, the SOD activity increased and remained at that high level until day 9 when it approximately doubled the value of activity noted for hypocotyls at 4 days of culture. The highest level of SOD activity was found in explants cultured for 14 days on RIM.

**Fig. 6:**
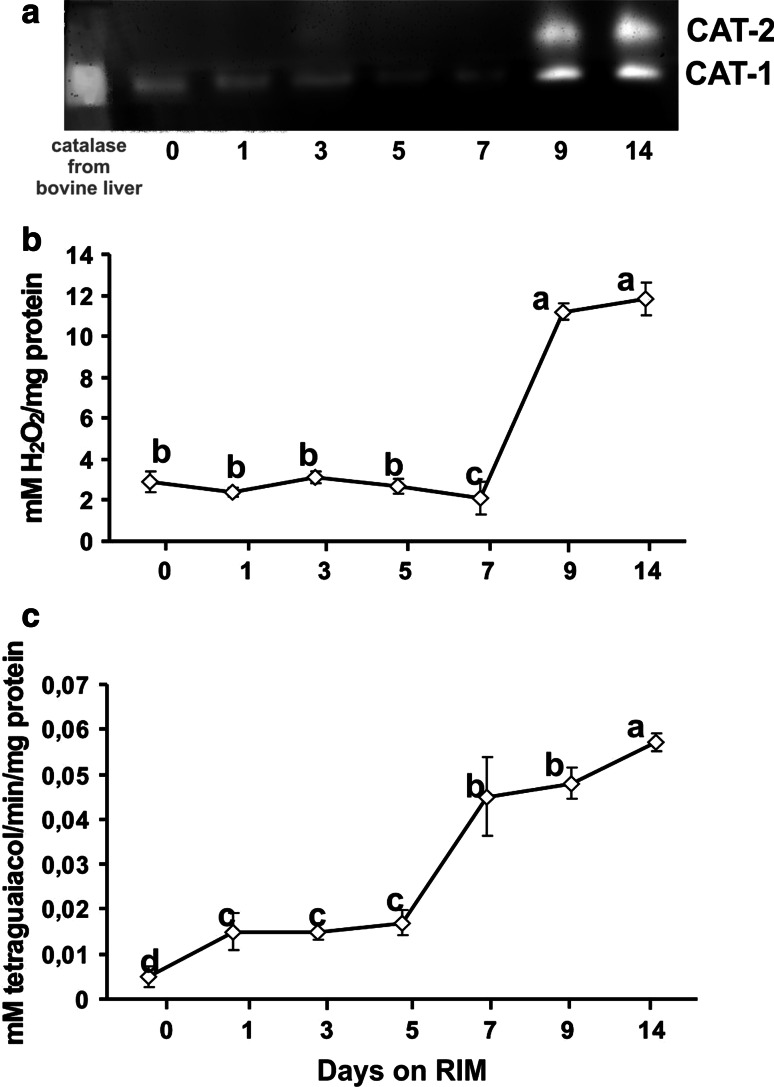
CAT (**a**, **b**) and guaiacol-dependent POX (**c**) activity patterns in explants maintained on RIM for 14 days. **a** Staining of CAT with ferricyanide following native PAGE of protein extracts. Each well was loaded with 7.5 μg proteins. Catalase from bovine liver (3,000 units/mg protein, Sigma–Aldrich) was loaded as a control. **b** Total CAT activity after spectrophotometric analysis. **c** Total guaiacol-dependent POX activity after spectrophotometric analysis. Values on graphs represent mean ± standard error (SE). Treatments bearing the *same letter* do not differ significantly (*p* ≤ 0.05) by Tukey’s multiple range test

Ferricyanide staining revealed the presence of two bands of CAT activity in the studied material: a fast-migrating band (called CAT-1 here) present in freshly cut hypocotyls and in explants throughout culture on RIM, and a slower-migrating one (CAT-2) expressed at days 9 and 14 of culture (Fig. 6a). Spectrophotometric analyses revealed no change in total CAT activity up to day 5 of culture on RIM, followed by a slight decline at day 7 (Fig. 6b). Then, the activity of CAT increased about fourfold by day 9 and remained at similar high levels through day 14.

One day after explantation on RIM, the activity of POX was slightly higher than in freshly cut hypocotyls and remained at that level until day 5 (Fig. 6c). Next, POX activity approximately tripled by day 7. Further culture witnessed a continuous increase in POX activity, which at day 14 reached levels about sixfold higher than in initial explants.

### Rhizogenesis is accompanied by conspicuous changes in PSII activity and oxygen uptake rate

**Fig. 7:**
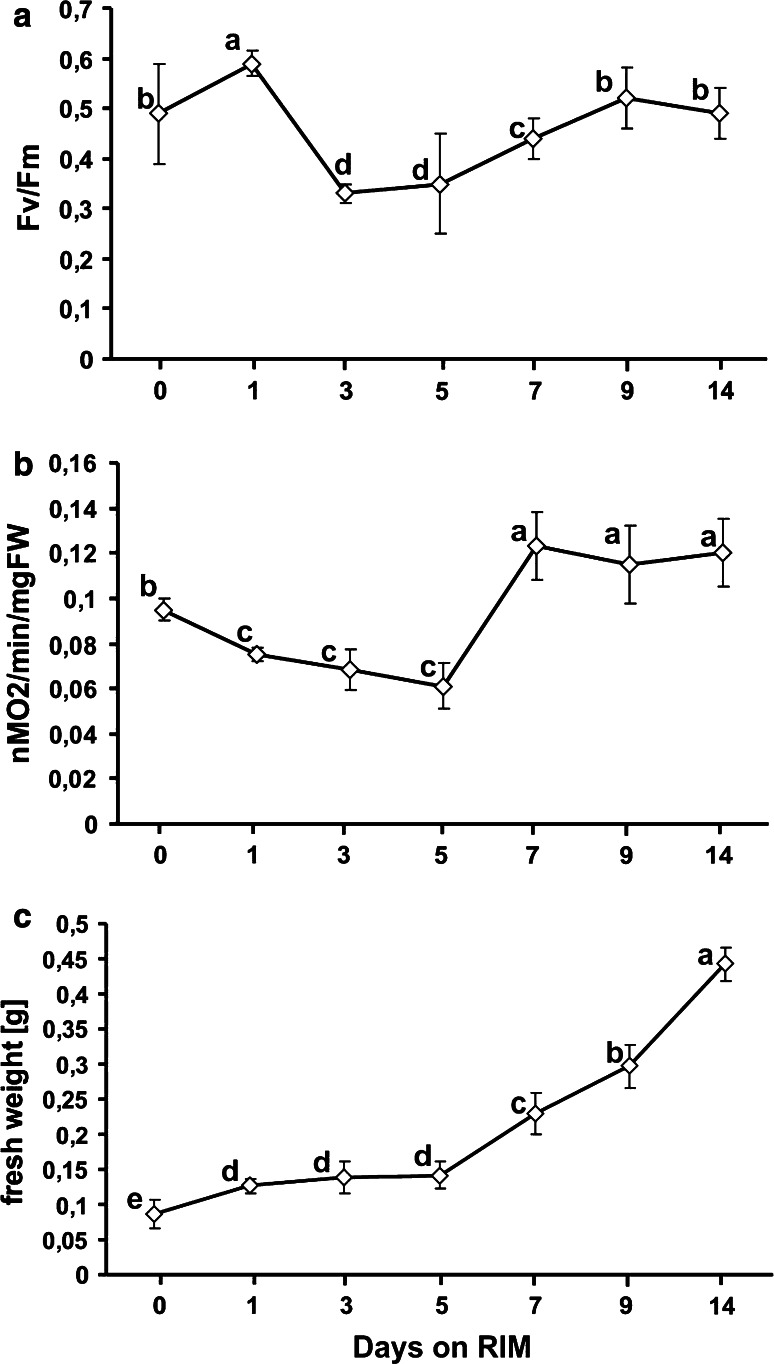
Maximum photochemical efficiency of photosystem II (**a**), rate of O_2_ uptake (**b**) and change in FW (**c**) of explants maintained for 14 days on RIM. Values on graph represent mean ± standard error (SE). Treatments bearing the *same letter* do not differ significantly (*p* ≤ 0.05) by Tukey’s multiple range test

PSII activity was the highest in explants maintained on RIM for 1 day (Fig. 7a). With continued culture it halved, bottoming on days 3 and 5 and then gradual increasing up to day 9. PSII activity levels in material cultured for 9 and 14 days and in hypocotyls before explantation were similar.

The rate of oxygen uptake decreased just after explantation and gradually declined until day 5 on RIM (Fig. 7b). At day 7 of culture, there was a conspicuous increase in oxygen uptake rate which remained unchanged through day 14.

Culture on RIM was accompanied by a gradual increase in explants fresh weight, most conspicuous between days 5 and 7 (Fig. 7c). At the end of the second week of culture, the mean FW of a single explant was about six times higher than for freshly cut hypocotyls.

### McGLP in rhizogenic cultures is not expressed

**Fig. 8:**
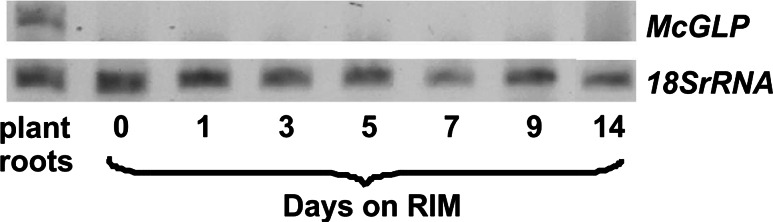
Expression of *McGLP* transcript in explants cultured for 14 days on RIM and in roots derived from 3-month-old plants growing in soil

*McGLP* was not observed in the material maintained on RIM. There was a clear band of *McGLP* transcript in material from roots of 3-month-old plants grown in soil (Fig. 8).

### AA inhibits rhizogenesis, lowers H_2_O_2_ content and changes SOD activity pattern

Adding AA to RIM reduced the frequency of root regeneration in a concentration-dependent manner. When RIM was supplemented with 2.5 μM AA, the hypocotyls produced three times fewer roots (mean 1.6 roots/explant) and the frequency of rhizogenesis (mean 21.6 %) was about half that of hypocotyls cultured on RIM alone (Fig. 1d). A concentration of 5 μM AA completely inhibited root formation; instead of roots, some explants produced very small amounts of non-regenerative callus from the cut end (Fig. 1e).

Histological sections through explants maintained on RIM with 5 μM AA did not reveal meristemoids. Within 1–4 days of culture on that medium, the general pattern of cell proliferation resembled that observed in explants cultured on RIM alone. Meristematic activity then expanded to the cortex, giving rise to endogenous callus of parenchymatous character, which ruptured the epidermis by day 10 (Fig. 3f, g).

Adding 5 μM AA to RIM had an evident effect on endogenous H_2_O_2_ content (Fig. 4). In explants maintained on such medium, the H_2_O_2_ level did not change until 9 days of culture when its concentration began to increase. Unlike RIM-cultured explants, no MnSOD-2 band was detected in hypocotyls cultured in the presence of 5 μM AA (Fig. 5b).

## Discussion

### Meristemoid formation coincides with induction of rhizogenic determination

This study characterized histological and biochemical features of direct rhizogenesis from *M. crystallinum* hypocotyl explants. To relate the biochemical and anatomical events accompanying root regeneration to developmental stages of rhizogenesis, we used tissue-transfer procedures which allowed us to experimentally define the temporal framework of organogenic competence and determination (Christianson and Warnick 1983, 1985). Our results show that to induce root production it was necessary to add 2,4-D to the medium, and that preculture on BM could shorten the time required for roots to appear on RIM by 3 days (Table 1; Fig. 2a, b), suggesting that during the first 3 days of culture on RIM, the explants were not competent to respond to the rhizogenic action of 2,4-D. This finding is in accord with previous studies which showed, in both direct and indirect regeneration, that cultured tissue may require a period of time to gain organogenic/embryogenic competence (Christianson and Warnick 1983, 1985; Attfield and Evans 1991; Finstad et al. 1993). In *M. crystallinum* hypocotyls, 5-day exposure to RIM was needed to induce a repeatable level of rhizogenesis, indicating that rhizogenic determination had begun in some of the explants. According to our histological studies, the onset of the determination state coincided with meristemoid production at the periphery of the vascular cylinder (Fig. 3d). This observation agrees with previous studies by Villalobos et al. (1985) and Flinn and Webb (1988) and allows us to identify the observed meristemoids as organized structures having a fixed developmental commitment. After determination has occurred, determined cells will continue to develop, eventually differentiating adventitious organs (Christianson and Warnick 1983, 1985). Indeed, the histology of explants maintained on RIM revealed the presence of fully developed root apices as early as 2 days after induction of rhizogenic determination (Fig. 3e). Hence, day 7 on RIM can be taken as the onset of the post-determination phase, which includes morphological differentiation of roots.

### Increase in endogenous H_2_O_2_ indicates the onset of rhizogenic competence

Acquisition of organogenic competence has been ascribed to the expression of specific regulatory genes, but the mechanisms controlling this phenomenon are poorly understood (Che et al. 2007). Previously we reported that *M. crystallinum* calli capable of rhizogenic and embryogenic development differed significantly in endogenous H_2_O_2_ content, suggesting the possible role of H_2_O_2_ in induction of these two alternative developmental pathways (Libik et al. 2005). Our present work revealed conspicuous changes in H_2_O_2_ levels during successive stages of rhizogenesis, with H_2_O_2_ content peaking at day 3 of culture on RIM (Fig. 4). A number of authors have reported an increase in endogenous H_2_O_2_ preceding differentiation of somatic embryos (Kairong et al. 1999; Dutta Gupta and Datta 2003; Li et al. 2007, 2009a Agrawal and Purohit 2012) under in vitro conditions. In those studies, however, the changes in H_2_O_2_ concentration were not related to the developmental milestones of competence, determination and morphological differentiation. In our work, H_2_O_2_ reached maximum at the time of acquisition of rhizogenic competence on RIM, that is, when the explants became responsive to the rhizogenic action of 2,4-D. H_2_O_2_ has been demonstrated to mediate the auxin response during the formation and development of lateral and adventitious roots (Li et al. 2007, 2009b; Tyburski et al. 2006). Our experiments revealed that adding 2.5 or 5 μM AA to RIM inhibited rhizogenesis in a concentration-dependent manner (Fig. 1d, e) and histological analysis of explants maintained on RIM with 5 μM AA made it clear that ascorbate prevented the formation of meristemoids (Fig. 3f, g); the explants from that medium had also much lower H_2_O_2_ content during early days of culture than the rhizogenic ones from RIM (Fig. 4). Thus, we can speculate that a certain level of H_2_O_2_ is required for meristemoid production (here, equivalent to induction of rhizogenic determination) and subsequent root development from *M. crystallinum* hypocotyls. An increase in endogenous H_2_O_2_ is needed to induce regeneration in tobacco (Siminis et al. 1994) and *Lycium barbarum* (Kairong et al. 1999). High endogenous H_2_O_2_ content in early stages of ice plant rhizogenesis coincided with low activities of the H_2_O_2_-decomposing enzymes CAT and guaiacol-dependent POX (Fig. 6a–c).

We also showed a transient but significant decrease in PSII activity not accompanied by changes in respiratory rate or explant FW within 1–5 days of culture on RIM (Fig. 7a–c). This seems to indicate that before meristemoid formation the explants were metabolically arrested. Such a transient “semi-dormant” state of cultured tissues followed by organ growth and development is considered a type of adaptive response to the stress imposed by in vitro culture (Gaspar et al. 2002).

### Decreased endogenous H_2_O_2_ content marks the onset of morphological differentiation of roots

Unlike the predetermination phase of rooting, the post-determination stage of the process in *M. crystallinum* was characterized by a very low endogenous H_2_O_2_ level along with high CAT and guaiacol-dependent POX activities (Figs. 4, 6a–c). The post-determination stage of rhizogenesis involves root growth and tissue maturation, two energy-consuming processes (Thorpe and Meier 1974; Prasad et al. 1994 Millar et al. 1998). Indeed, with the onset of the post-determination phase, PSII activity as well as the respiratory rate increased significantly versus the early stages of culture (Fig. 7a, b). The close relationship between the rise in FW of explants and the activity of guaiacol-dependent POX seems to confirm the well-known function of this enzyme in cell wall formation and modification associated with organ growth and tissue maturation (Bonfill et al. 2003; Lepeduś et al. 2004; Molassiotis et al. 2004). During rhizogenesis in *Clerodendrum viscosum*, intensification of carbohydrate catabolism was accompanied by a conspicuous increase in CAT activity (Prasad et al. 1994). We noted a similar relationship in this study. The increased CAT activity can be attributed to induction of an additional form, CAT-2, which occurred at day 9 of culture on RIM (Fig. 6a). Increased activity of root-specific CAT forms has been observed in several species including maize (Chandlee et al. 1983; Racchi et al. 2001; Verma and Dubey 2003). In *M. crystallinum*, however, the occurrence of the CAT-2 band does not seem related to rooting itself, as it has been found in fast-growing but non-regenerative or embryogenic calli (Libik et al. 2005). In view of that finding, CAT-2 expression in direct rhizogenesis can be accounted for by the high rate of oxidative metabolism and the need for an additional antioxidative defense during the growth of newly produced organs.

### Induction of specific MnSOD form marks the onset of determination and rhizogenic development

Electrophoretic analysis of SODs revealed significant changes in the pattern of isoenzymes: an additional band of MnSOD-2 activity was observed within 5–14 days of culture on RIM (Fig. 5a). The occurrence of the MnSOD-2 band in *M. crystallinum* has been suggested to be associated with the low photosynthetic activity of cultured tissue (Libik et al. 2005) and/or the respiration-stimulating conditions of in vitro culture (Ślesak and Miszalski 2002). However, our study found no relationship between the expression of MnSOD-2 activity and the metabolic status of the explants: the MnSOD-2 band appeared in early rhizogenesis when the explants showed low O_2_ uptake and PSII activity, but it was also maintained through the entire post-determination phase, during which the rate of carbohydrate metabolism was relatively high. Induction of root-specific SOD forms has been reported in several plant species, and usually ascribed to the response of the assayed organs to different stress stimuli (Chongpraditnum et al. 1992; Przymusiński et al. 1995; Lambais et al. 2003). In *M. crystallinum,* the MnSOD-2 band was not detected in material from explants maintained in the presence of AA (Fig. 5b), so the antioxidative function of this protein cannot be ruled out. When considering the possible role of MnSOD-2 in plant physiology we need to keep in mind its specific biochemical characteristics, such as extreme thermal stability, resistance to detergents and relatively high molecular mass (Ślesak and Miszalski 2002), which call into question the SOD-like nature of this protein. Ślesak and Miszalski (2002) suggested that MnSOD-2 from *M. crystallinum* roots may in fact represent a GLP having SOD activity. Indeed, GLPs from different plant species have been shown to display SOD-type activity (Carter and Thornburg 2000; Christensen et al. 2004; Guicciardo et al. 2007), and root-specific *GLP* mRNA was identified in *M. crystallinum* from hydroponic (Michalowski and Bohnert 1992). Our study did not confirm the putative germin-like character of MnSOD-2, however, as no *McGLP* transcript was identified in the explants maintained on RIM (Fig. 8). The occurrence of the MnSOD-2 band exactly at the onset of rhizogenic determination—and its maintenance throughout the course of rhizogenesis on RIM—links it specifically to rooting and allows us to consider it a molecular marker of rhizogenesis in *Mesembryanthemum crystallinum*. The chemical nature and biological role of MnSOD-2 protein in root ontogeny remain to be elucidated.

## Abbreviations

AA: l-Ascorbic acid
BM: Basal medium
BSA: Bovine serum albumin
CAT: Catalase (EC 1.11.1.6)
DTT: Dithiothreitol
2,4-D: 2,4-Dichlorophenoxy-acetic acid
EDTA: Ethylenediaminetetraacetic acid
Fm: Maximum chlorophyll *a* fluorescence
FW: Fresh weight
*F*_v_: Variable fluorescence of chlorophyll *a*
*F*_v_/*F*_m_: Maximum photochemical efficiency of photosystem II
GLP: Germin-like protein
NBT: Nitro blue tetrazolium
PAGE: Polyacrylamide gel electrophoresis
POX: Peroxidase (EC 1.11.1.7)
RIM: Root induction medium
ROS: Reactive oxygen species
SDS: Sodium dodecyl sulfate
SOD: Superoxide dismutase (EC 1.15.1.1)
TEMED: *N*,*N*,*N*′,*N*′-Tetramethylethylenediamine
TRICINE: *N*-tris[Hydroxymethyl]methylglycine
TRIS: tris(Hydroxymethyl)aminomethane
